# Transfer‐function‐free technique for the noninvasive determination of the human arterial pressure waveform

**DOI:** 10.14814/phy2.15040

**Published:** 2021-09-22

**Authors:** Alessandro Giudici, Carlo Palombo, Carmela Morizzo, Michaela Kozakova, J. Kennedy Cruickshank, Ian B. Wilkinson, Ashraf W. Khir

**Affiliations:** ^1^ Department of Mechanical and Aerospace Engineering Brunel University London Uxbridge UK; ^2^ Department of Surgical Medical, Molecular Pathology and Critical Area Medicine University of Pisa Pisa Tuscany Italy; ^3^ Department of Clinical and Experimental Medicine University of Pisa Pisa Tuscany Italy; ^4^ School of Life‐Course/Nutritional Sciences King’s College St. Thomas’ & Guy’s Hospitals, London Middlesex UK; ^5^ Division of Experimental Medicine and Immunotherapeutics University of Cambridge Cambridge Cambridgeshire UK

**Keywords:** carotid artery, hypertension, local wave speed, noninvasive estimation of pressure, pressure

## Abstract

The estimation of central aortic blood pressure is a cardinal measurement, carrying effective physiological, and prognostic data beyond routine peripheral blood pressure. Transfer function‐based devices effectively estimate aortic systolic and diastolic blood pressure from peripheral pressure waveforms, but the reconstructed pressure waveform seems to preserve features of the peripheral waveform. We sought to develop a new method for converting the local diameter distension waveform into a pressure waveform, through an exponential function whose parameters depend on the local wave speed. The proposed method was then tested at the common carotid artery. Diameter and blood velocity waveforms were acquired via ultrasound at the right common carotid artery while simultaneously recording pressure at the left common carotid artery via tonometer in 203 people (122 men, 50 ± 18 years). The wave speed was noninvasively estimated via the ln*DU*‐loop method and then used to define the exponential function to convert the diameter into pressure. Noninvasive systolic and mean pressures estimated by the new technique were 3.8 ± 21.8 (*p* = 0.015) and 2.3 ± 9.6 mmHg (*p* = 0.011) higher than those obtained using tonometery. However, differences were much reduced and not significant in people >35 years (0.6 ± 18.7 and 0.8 ± 8.3 mmHg, respectively). This proof of concept study demonstrated that local wave speed, estimated from noninvasive local measurement of diameter and flow velocity, can be used to determine an exponential function that describes the relationship between local pressure and diameter. This pressure‐diameter function can then be used for the noninvasive estimation of local arterial pressure.

## Graphical abstract

The noninvasive determination of local arterial pressure is of great interest to both clinicians and physiologists. In this work we present a novel technique for the determination of arterial pressure using direct and noninvasive local measurements. The results are encouraging and warrant further investigation to establish the full extent of benefits of this method.

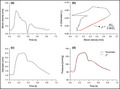

## 1 INTRODUCTION

The World Health Organization estimates that ~15% of the population worldwide suffers from high blood pressure, and only 20% of these are effectively managing their condition. According to the European Society of Hypertension, the current definition of hypertension entails having a brachial systolic blood pressure (*P*_s_) ≥140 mmHg and/or diastolic blood pressure (*P*_d_) ≥90 mmHg (Williams et al., 2018). In contrast to central aortic pressure, which may only be accurately measured invasively, brachial *P*_s_ and *P*_d_ can easily be assessed noninvasively via cuff measurement and are the gold standard in daily clinical practice because, for nearly 100 years, each was consistently shown to predict adverse cardiovascular outcomes.

Mean blood pressure (*P*_m_) and *P*_d_ and are relatively constant throughout most of the arterial tree (Pauca et al., 2001; Wang et al., 2011), but *P*_s_ increases as the measurement site moves distally from the ascending aorta (Reference Values for Arterial Measurements Collaboration, 2014; Segers et al., 2009), most likely due to wave reflections and higher wall stiffness in the distal arteries compared to the aorta. Further, the magnitude of the pressure amplification is age, sex, and pathology dependent (McEniery et al., 2014; Reference Values for Arterial Measurements Collaboration, 2014). Therefore, using brachial pressure to estimate pressure in other regions of the arterial tree will generally be poor (Sharman et al., 2017).

Several studies have shown the added predictive value of central blood pressure for future cardiovascular events and stroke mortality, beyond brachial pressure and independent from established cardiovascular risk factors (Cheng et al., 2013; Chirinos et al., 2013). Indeed, it is expected that central blood pressure reflects the hemodynamic load on the left ventricle more accurately than brachial pressure (Roman et al., 2007). Further, central and peripheral blood pressure can be differentially affected by antihypertensive therapy, with potential clinical implications on patients management of hypertension and heart failure (Borlaug et al., 2014; Sharman et al., 2013; Williams et al., 2006). These findings promoted the development of methods to noninvasively estimate aortic pressure.

Transfer function‐based techniques are currently the most commonly used methods for the noninvasive determination of aortic pressure. These functions describe the relationship between the central aortic pressure and the pressure measured at a peripheral site, and can be used to convert peripheral pressure waveforms into central aortic pressure (Costello et al., 2015; Ghasemi et al., 2017). While several commercial devices have been produced to solve this task (Ding et al., 2011) and empirical evidence suggests that some devices accurately predict the *P*_s_–*P*_d_ range in the aorta (Ding et al., 2011), the estimated waveforms are similar to and preserve features of the measured peripheral pressure waveform (Millasseau et al., 2003; Segers et al., 2005). Hence, the waveforms estimated by the transfer functions might better represent the peripheral waveform than aortic root waveforms.

To overcome this issue, alternative methods have been introduced to directly estimate pressure from local arterial waveforms acquired noninvasively and a local estimate of wave speed (*c*) (Beulen et al., 2011; Vennin et al., 2015). Similar to transfer functions, these methods typically rely on the assumption that *P*_d_ and *P*_m_ are the same in most arterial locations. Vennin et al. (2015) proposed a method to reconstruct the aortic pressure waveform (*P*) from noninvasive acquisition of aortic blood flow velocity waveform (*U*), peripheral *P*_s_ and *P*_d_, and features of the exponential decay of peripheral pressure in diastole. The method relies on values of *c* and the water hammer equation (Khir et al., 2001) to convert the systolic ejection in the velocity waveform into the pressure upstroke. While this method provided plausible estimations of aortic *P* both in computational and in vivo settings, the pressure waveform involved in the determination of *c* (Davies et al., 2005) was recorded invasively. This renders the technique unsuitable for routine examination, although its relative accuracy is yet to be determined if *c* is estimated noninvasively. Beulen et al. (2011) used simultaneous ultrasound measurements of *U* and diameter distension waveform (*D*) to estimate *P* in flexible tubes. The flow‐area (*Q*–*A*) method (Rabben et al., 2004) was used to determine *c* noninvasively, and the relationship between *c* and distensibility (*Ds*), described by the Bramwell–Hill equation (Bramwell et al., 1923), was used to calculate pressure by integrating changes in tube cross‐sectional area with respect to the diastolic reference. This method assumes that *c* is constant across the pressure range of the entire cardiac cycle. While this assumption might be correct in the case of flexible tubes with a linear *P*–*A* relationship, the latter is nearly exponential in arteries (Gavish & Izzo, 2016), implying that *c* does increase with increasing pressure.

The aim of this study was to develop a noninvasive method for estimating arterial pressure from local hemodynamic waveforms. Our ultimate goal was to provide an alternative to transfer functions using local noninvasive measurements for estimating central aortic pressure. To that end and as a proof of concept, we tested the new technique using data measured at the common carotid artery (CCA) and compared the results against applanation tonometry.

## 2 METHODS

The general methodology of the technique is to construct the exponential relationship between *P* and *A* in arteries using local *c*, which can be determined noninvasively from ultrasound measurement of local *D* and *U* using the ln*DU*‐loop method (Feng & Khir, 2010). Once the *P*–*A* relationship is established, *P* is estimated using noninvasive local measurement of *D* and peripheral *P*_d_. We applied this approach to carotid artery data, comparing the new technique with applanation tonometry, a well‐established method for the recording of pressure in superficial arteries (Segers et al., 2009).

### 2.1 Theoretical background

Tube laws describe the relationship between *P* and *A*, or *D* of a flexible tube. Assuming that arteries are cylindrical, the *P*–*A* relationship of arteries, closely resembling an exponential function (Fung, 1967; Spronck et al., 2015), can be written in terms of *P* and *D* ^2^. The tube law used in this study is that proposed by Meinders and Hoeks (2004) (Equation 1):

(1) $$P\left(D\right)=P_{d}\cdot e^{\gamma \left(\frac{D^{2}}{D_{d}^{2}}‐1\right)},$$

where *P*_d_ is the diastolic pressure, *D*_d_ is the diastolic diameter (i.e., the diameter at *P*_d_), and *γ* is an exponential gain defining the relationship between *P* and *D* ^2^. The objective of the following derivation is to re‐write Equation 1 using noninvasive parameters; thus allowing for the noninvasive determination of pressure.

Arterial distensibility is defined as *Ds* = d*A*/(*A*d*P*), where d*A* is the change in the vessel cross‐sectional area in response to a change in pressure (d*P*). The relationship between *Ds* and *c* is expressed in the Bramwell–Hill equation (Equation 2) (Bramwell et al., 1923):

(2) $$c=D\sqrt{\frac{dP}{\rho \cdot d\left(D^{2}\right)}},$$

where *ρ* is the blood density. Equation 2 states that the wave speed *c* at any given pressure *P_c_* can be expressed as a function of the slope of the tangent to the *P*–*D* ^2^ relationship at the pressure level *P_c_* (and corresponding *D_c_* so that *P* (*D_c_*) = *P_c_*). Therefore, for any estimate of *c* there must be a *P_c_* satisfying Equation 2. Using Equation 1, the derivative term of Equation 2 can be rearranged as

(3) $$\frac{dP}{dD^{2}}=\frac{\gamma \cdot P_{d}}{D_{d}^{2}}e^{\gamma \left(\frac{D^{2}}{D_{d}^{2}}‐1\right)}.$$

Inverting Equation 1 to express the diameter as a function of pressure, we obtain

(4) $$D\left(P\right)=D_{d}\sqrt{1+\frac{\ln\left(\frac{P}{P_{d}}\right)}{\gamma }},$$

and substituting *D* in Equation 3 with Equation 4, the derivative term is furtherly manipulated in:

(5) $$\frac{dP}{dD^{2}}=\frac{\gamma \cdot P_{d}}{D_{d}^{2}}\cdot e^{\gamma \left[\frac{\ln\left(\frac{P}{P_{d}}\right)}{\gamma }\right]}=\frac{\gamma \cdot P_{d}}{D_{d}^{2}}\cdot e^{\ln\left(\frac{P}{P_{d}}\right)}=\frac{\gamma \cdot P}{D_{d}^{2}}.$$

Substituting Equation 5 into Equation 2 and knowing that for any given *c* Equation 2 is verified for *P* = *P_c_*, we obtain:

(6) $$c^{2}=\frac{D_{c}^{2}\cdot \gamma \cdot P_{c}}{D_{d}^{2}\cdot \rho }.$$

Then, replacing *D_c_* in Equation 6 with Equation 4 and solving for *γ* leads to the following relationship:

(7) $$\gamma =\frac{\rho \cdot c^{2}}{P_{c}}‐\ln\left(\frac{P_{c}}{P_{d}}\right),$$

where *P_c_* is the pressure level at which *c* is calculated. Hence, it is expected that *P_c_* is the representative pressure for pressure range pertaining to the method chosen for the estimation of *c*.

### 2.2 Study population and acquisition protocol

The data used in this study were acquired at the University Hospital of Pisa. The study population included 203 people (122 men, 51±17 years, age range 16–78 years) undergoing standard out‐patient cardiovascular risk assessment, all free of major cardiovascular events, atrial fibrillation, malignancy, or chronic inflammatory disease. All subjects were referred for a complete cardiovascular examination to the Clinic for Cardiometabolic Risk Prevention of the Department of Surgical and Medical Pathology, University of Pisa. The protocol of the study followed the principles of the Declaration of Helsinki and was approved by the institutional ethics committee “Comitato Etico di Area Vasta Nord Ovest” (reference number: 3146/2010). Everyone gave their informed consent to participate. Clinical characteristics of the study participants are reported in Table S1 (https://figshare.com/s/4aab7f7fd026d8fbb761).

*P*, *D*, and *U* waveforms of the CCAs were acquired simultaneously by a single experienced operator (C.M.), following an earlier reported protocol (Giannattasio et al., 2008). Simultaneous ultrasound acquisition of *D* and *U* was performed on the right CCA using a 10.0‐MHz linear array probe with radiofrequency data output at the frequency of 1 kHz connected to an Aloka Alpha10 Prosound system (Hitachi Ltd., Tokyo, Japan) as reported previously (Giudici et al., 2020). Given the impossibility of placing a pressure tonometer on the right CCA due to the presence of the ultrasound probe, *P* was acquired at the left CCA using a PulsePen (DiaTecne, Milan, Italy) with sampling frequency 1 kHz. Acquisitions lasted for approximately 10 s, granting at least seven heartbeats where *P* and *D*‐*U* were recorded simultaneously.

The carotid ultrasound/tonometer acquisitions were performed after the subject had rested in the supine position for at least 10 min. Brachial *P*_s_ and *P*_d_ (_b_ *P*_s_ and _b_ *P*_d_) were measured by an electronic digital manometer (Omron, model 705cp, Kyoto, Japan) and the average of two consecutive measurements was used for calibration.

The data that support the findings of this study are available from the corresponding author upon reasonable request.

### 2.3 Noninvasive estimation of the local wave speed (_n_ *c*) and exponential gain (_n_ *γ*)

**FIGURE 1:**
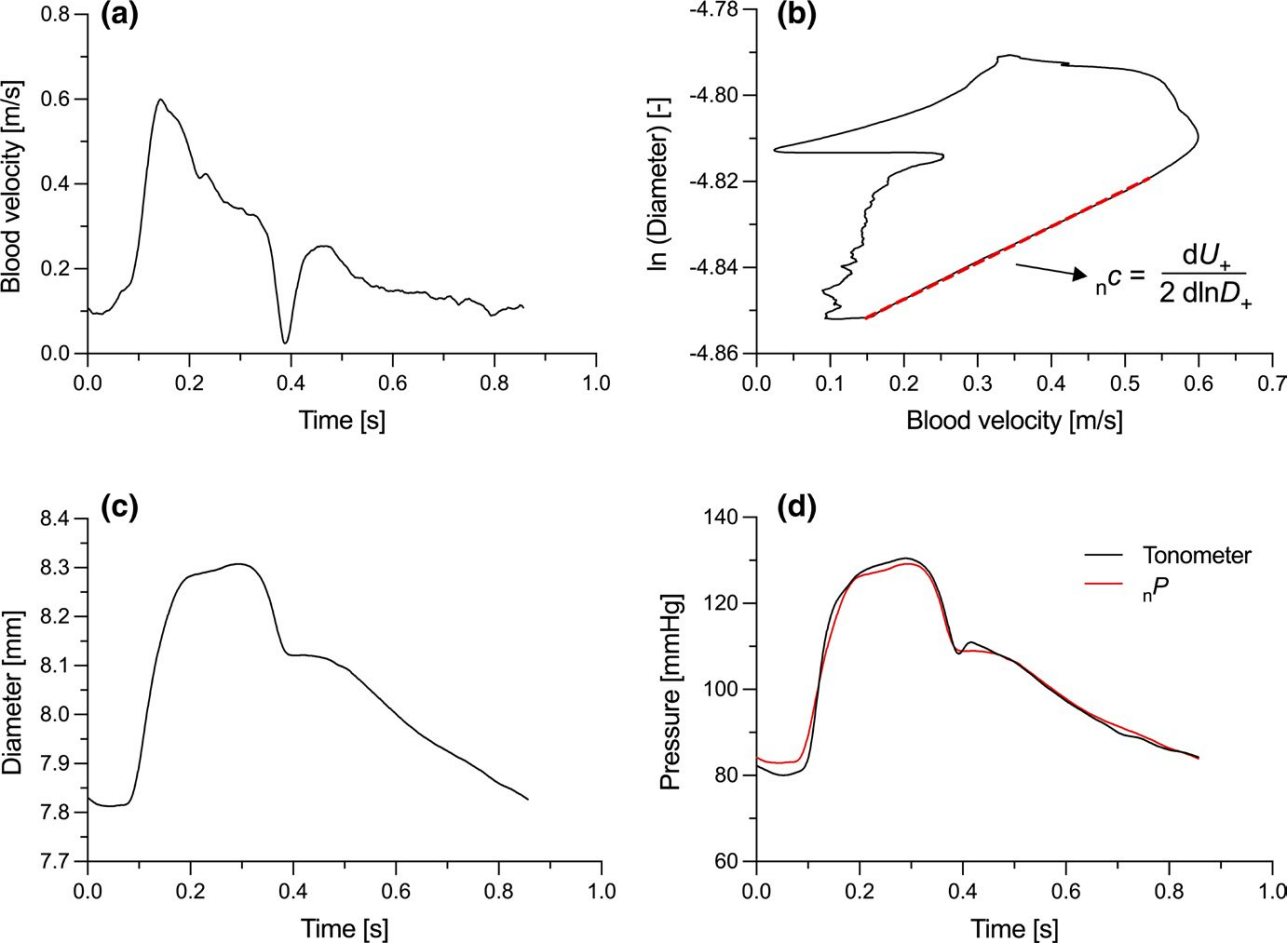
Ensemble averaged measurements of blood flow velocity (*U*) (a) and diameter (*D*) (c) waveforms measured at the carotid artery for a 74 years old patient. The ln*DU*‐loop and noninvasive pressure (_n_ *P*) estimation are presented, respectively in (b, d). The noninvasive wave speed (_n_ *c*) is estimated from the slope of the initial linear part of the ln*DU*‐loop (Equation 8). _n_ *c* is then used to estimate the exponential gain _n_ *γ* (Equation 9) and convert the diameter waveform into a pressure waveform using Equation 11

The local CCA wave speed was estimated using the ln*DU*‐loop method, whose complete derivation is described in earlier work (Feng & Khir, 2010). Briefly, when arterial waves are unidirectional (i.e., travelling only from the heart toward the periphery or vice versa), the relationship between the *U* and the natural logarithm of *D* is linear and proportional to the wave speed (Equation 8).

(8) $${}_{n}c=\pm \frac{1}{2}\frac{dU_{\pm }}{d(\ln D_{\pm })},$$

where subscripts + and − indicate forward (from the heart to the periphery) and backward (from the periphery to the heart) direction of wave travel. Following cardiac ejection, a forward travelling compression wave is generated. Assuming only forward waves exist in early systole, as it will be too early for reflected waves to return from the periphery, the unidirectionality of the waves is reasonable, and _n_ *c* can be determined with Equation 8 (Figure 1b).

The exponential gain _n_ *γ* can then be calculated using Equation 7 and _n_ *c* if *P_c_* for the ln*DU* method is known. Given the inherent assumption in the loop methods that *c* is constant during that pressure range, we take *P*_d_ as fiducial marker of the early systolic pressure range that is easily obtained noninasively since constant throughout the circulation. Hence, we assume that *P_c_* = *P*_d_ = _b_ *P*_d_ and Equation 7 reduces to

(9) $${}_{n}\gamma =\frac{\rho \cdot {}_{n}c^{2}}{{}_{b}P_{d}}.$$

with *ρ* = 1060 kg/m^3^. A similar approach has been described previously for regional pulse wave velocity (Spronck et al., 2017).

To provide a means of comparison for _n_ *γ*, the exponential gain was also calculated using the tonometer waveform; inverting Equation 1 and considering the systolic pressure and diameter leads to

(10) $${}_{t}\gamma =\frac{\ln\left(\frac{{\;}_{\text{t}}P_{s}}{{\;}_{\text{t}}P_{d}}\right)}{\frac{D_{s}^{2}}{D_{d}^{2}}‐1},$$

where _t_ *P*_s_ and _t_ *P*_d_ are the average *P*_s_ and *P*_d_ of all the heartbeats (*N* = 7–10) of the tonometer acquisition (i.e., the peak and the minimum pressure in each cardiac cycle), and *D*_s_ and *D*_d_ are the average systolic and diastolic *D* determined from the ultrasound acquisition.

### 2.4 Noninvasive estimation of pressure

Assuming a uniform *P*_d_ throughout the arterial system, the diameter waveform can be converted into a pressure waveform using Equation 1 and _n_ *γ*.

(11) $${}_{n}P\left(D\right)={}_{b}P_{d}\cdot e^{{}_{n}\gamma \left(\frac{D^{2}}{D_{d}^{2}}‐1\right)}$$

As _b_ *P*_d_, *D* and _n_ *γ* are all determined noninvasively, _n_ *P* can therefore be determined entirely noninvasively. _n_ *P*_s_ was calculated as the average of the peaks of all the cardiac cycles (*N* = 7–10) of the estimated _n_ *P* waveform. _n_ *P*_m_ was the arithmetic mean of all the data points of _n_ *P*.

### 2.5 Statistical analysis

Data are reported as mean ± standard deviation (SD). _n_ *P*_s_ and _n_ *P*_m_ were compared with tonometer measurements of _t_ *P*_s_ and _t_ *P*_m_. The tonometer acquisition of pressure was calibrated using _b_ *P*_d_ and _b_ *P*_m_ and assuming constant *P*_m_ and *P*_d_ throughout the arterial system. _b_ *P*_m_ was estimated using a form factor (*FF*) of 0.43 as _b_ *P*_m_ = _b_ *P*_d_ + 0.43 (_b_ *P*_s_ − _b_ *P*_d_) (Segers et al., 2009). The noninvasive carotid *FF* (_n_ *FF*) was used as an additional parameter to quantify the accuracy of the new technique for estimating the pressure waveform ${}_{n}FF=\frac{{}_{n}P_{m}‐{}_{b}P_{d}}{{}_{n}P_{s}‐{}_{b}P_{d}}$, and compared to *FF* calculated from the left CCA tonometry (_t_ *FF*).

Comparison between noninvasive and tonometer output variables was initially performed using paired sample *t*‐test and Bland–Altman plots (Bland & Altman, 1986), first on the entire cohort and then stratifying the population in three age groups: young (<35 years), middle‐aged (35–59 years), and older adults (≥70 years). This allowed a first evaluation of the effect of age on the accuracy of the pressure estimation.

Then, age was used as a continuous variable in multivariate regression analysis, including _n_ *P* as dependent variable and corresponding _t_ *P* value (i.e., *P*_s_ and *P*_m_ independently), age, type 1 diabetes mellitus (T1DM), type 2 diabetes mellitus (T2DM), antihypertensive treatment, and dyslipidemia as independent variables.

Linear regression and correlation analysis were performed where appropriate. *p* ≤ 0.05 was considered statistically significant.

## 3 RESULTS

The hemodynamic characteristics of subjects included in this study are reported in Table 1. _b_ *P*_s_ and _b_ *P*_d_ were 122.1 ± 16.8 mmHg and 75.2 ± 10.3 mmHg, respectively. Using a form factor 0.43 (Equation 11) lead to _b_ *P*_m_ =95.4 ± 12.0 mmHg.

**TABLE 1:** Measured and noninvasively estimated hemodynamic characteristics of the people included in the study

	All	≤35 years	36–59 years	≥60 years
*N* (male)	203 (60%)	47 (59%)	78 (53%)	78 (68%)
Age [years]	51 ± 17	24 ± 5	51 ± 6	67 ± 5
Brachial artery				
_b_*P*_s_ [mmHg]	122.1 ± 16.8	111.4 ± 11.7	120.9 ± 14.7	129.8 ± 17.6
_b_*P*_d_ [mmHg]	75.2 ± 10.3	67.0 ± 8.5	77.8 ± 9.4	77.5 ± 9.5
Carotid artery				
_n_*c* [m/s]	5.67 ± 1.45	4.45 ± 0.73	5.58 ± 1.12	6.49 ± 1.50
_t_*γ* [–]	3.53 ± 1.48	2.11 ± 0.54	3.29 ± 0.99	4.64 ± 1.43
_n_*γ* [–]	3.60 ± 1.75	2.43 ± 0.80^†^	3.33 ± 1.30	4.53 ± 1.98
_t_*P*_s_ [mmHg]	120.3 ± 17.3	110.5 ± 13.0	118.5 ± 15.0	128.1 ± 18.1
_n_*P*_s_ [mmHg]	124.1 ± 23.6*	122.2 ± 25.6^†^	120.4 ± 18.9	127.5 ± 23.3
_t_*P*_m_ [mmHg]	95.4 ± 12.0	86.1 ± 8.4	96.3 ± 11.0	100.0 ± 11.7
_n_*P*_m_ [mmHg]	97.6 ± 13.6*	91.7 ± 12.1^‡^	97.6 ± 11.8	100.3 ± 13.7
_t_*FF* [–]	0.45 ± 0.04	0.45 ± 0.03	0.46 ± 0.04	0.45 ± 0.04
_n_*FF* [–]	0.46 ± 0.03^‡^	0.46 ± 0.03	0.47 ± 0.03*	0.46 ± 0.03^†^

Comparison between tonometry and new method: *p < 0.05, ^†^ *p* < 0.01, and ^‡^ *p* < 0.001.

Abbreviations: _b_ *P*_d_, brachial diastolic blood pressure; _b_ *P*_s_, brachial systolic blood pressure; _n_ *c*, noninvasive wave speed; _n_ *P*_m_, estimated carotid mean pressure; _n_ *P*_s_, estimated carotid systolic pressure; _n_ *γ*, exponential gain estimated from _n_ *c*; _t_ *FF* = (_t_ *P*_m_ − _b_ *P*_d_)/(_t_ *P*_s_ − _b_ *P*_d_), _n_ *FF* = (_n_ *P*_m_ − _b_ *P*_d_)/(_n_ *P*_s_ − _b_P_d_); _t_ *P*_m_, tonometer carotid mean blood pressure; _t_ *P*_s_, tonometer carotid systolic blood pressure; _t_ *γ*, exponential gain estimated from the tonometer pressure and ultrasound diameter waveforms.

**FIGURE 2:**
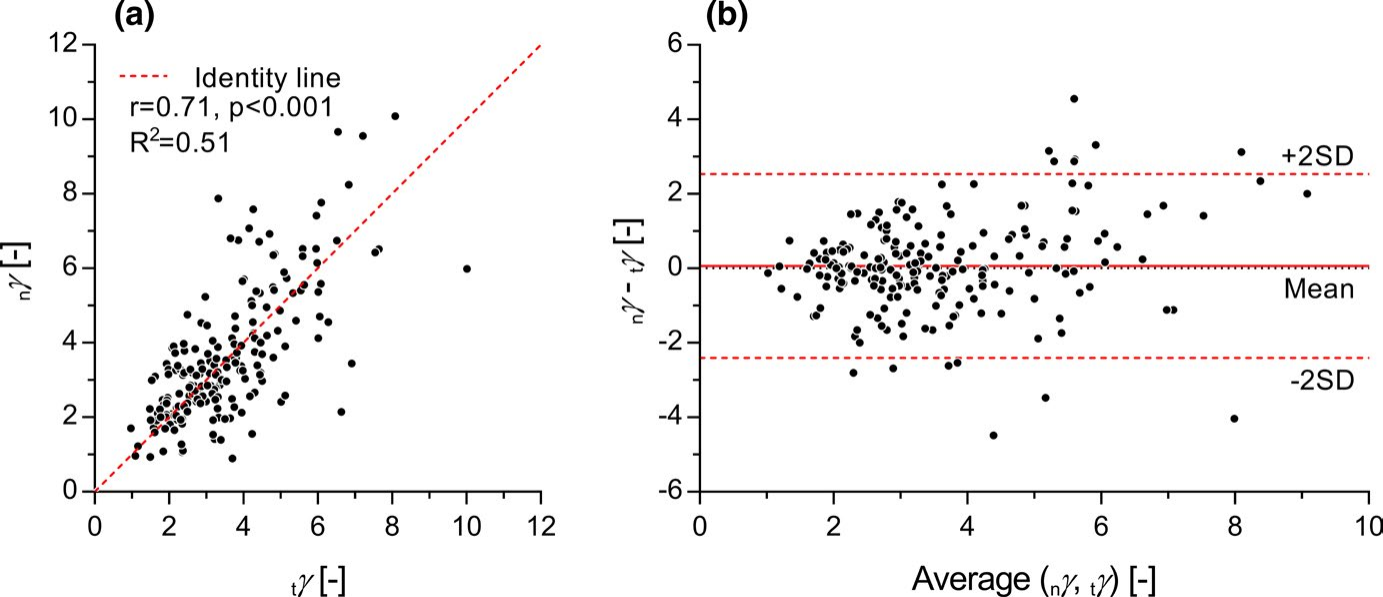
Correlation (a) and Bland–Altman (b) plot between exponential constants estimated from the tonometer pressure, _t_ *γ* (Equation 10), and from the noninvasive wave speed, _n_ *γ* (Equation 9), of the *N* = 203 subject included in this study. Limits of agreement were calculated as ±2 standard deviations (SD)

Average noninvasive wave speed _n_ *c* was 5.67 ± 1.45 m/s. _n_ *γ* was comparable to _t_ *γ* estimated using applanation tonometry (3.60 ± 1.75 vs. 3.53 ± 1.48, limits of agreement: −2.42 to 2.54, *p* = 0.49) and the two metrics showed strong correlation (Figure 2). When stratifying our cohort in age groups, _n_ *γ* was significantly higher than _t_ *γ* in young people (≤35 years, *p* = 0.006), but not in middle‐aged and older adults (*p* = 0.73 and *p* = 0.51, respectively) (Table 1 and Figure S1, https://figshare.com/s/4aab7f7fd026d8fbb761). However, in the multivariate regression analysis, no significant interaction was found between age and _n_ *γ* (*β* = 0.056, 95% confidence interval (CI) [−0.093–0.196], *p* = 0.47) (Table S2, https://figshare.com/s/4aab7f7fd026d8fbb761).

**FIGURE 3:**
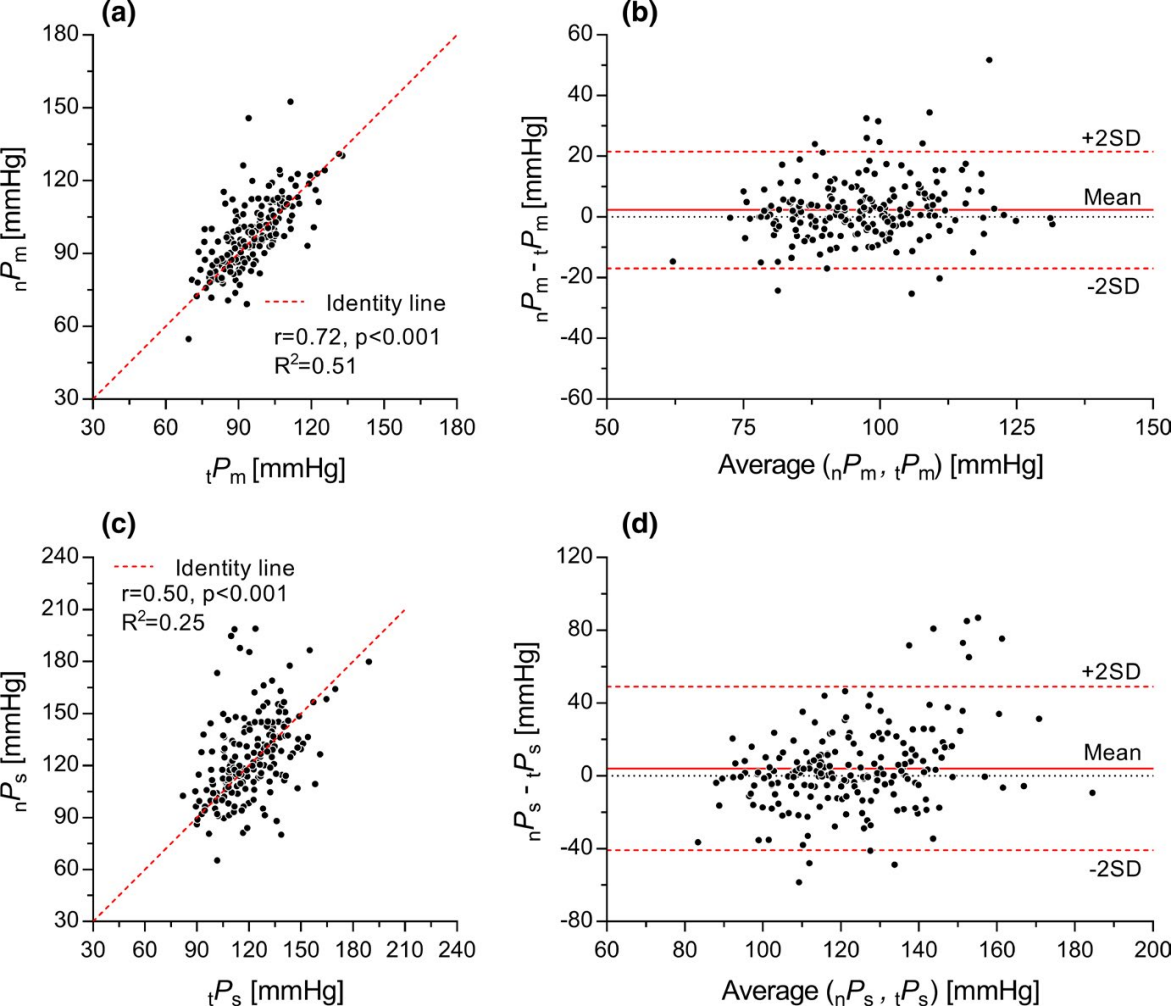
Correlation (a, c) and Bland–Altman (b, d) plots between tonometer pressure and pressure estimated with the new technique in the *N* = 203 subject included in this study: mean pressure (*P*_m_) (a, b) and systolic pressure (*P*_s_) (c, d). Limits of agreement were calculated as ±2 standard deviations (SD)

Figure 1d shows the comparison between _n_ *P*, estimated by the new technique, and *P*, acquired with tonometry, for a 74 year old subject included in this study. On average, noninvasive _n_ *P*_s_ and _n_ *P*_m_ were 3.8 (limits of agreement: −39.9 to 47.4) mmHg (*p* = 0.015) and 2.3 (−17.0 to 21.5) mmHg (*p* = 0.011) higher than _t_ *P*_s_ and _t_ *P*_m_ acquired via tonometry, respectively (Table 1 and Figure 3b–d). Correlation between the two techniques was strong for *P*_m_ (Figure 3a) and moderate for *P*_s_ (Figure 3c). Further, the Bland–Altmann plots (Figure 3b–d) showed weak correlations between the difference and average of *P*_m_ and *P*_s_ determined with the two techniques (*r* = 0.35, *p* < 0.001 and *r* = 0.18, *p* = 0.010, respectively). Overall, _n_ *FF* was slightly higher than _t_ *FF* (*p* < 0.001).

**FIGURE 4:**
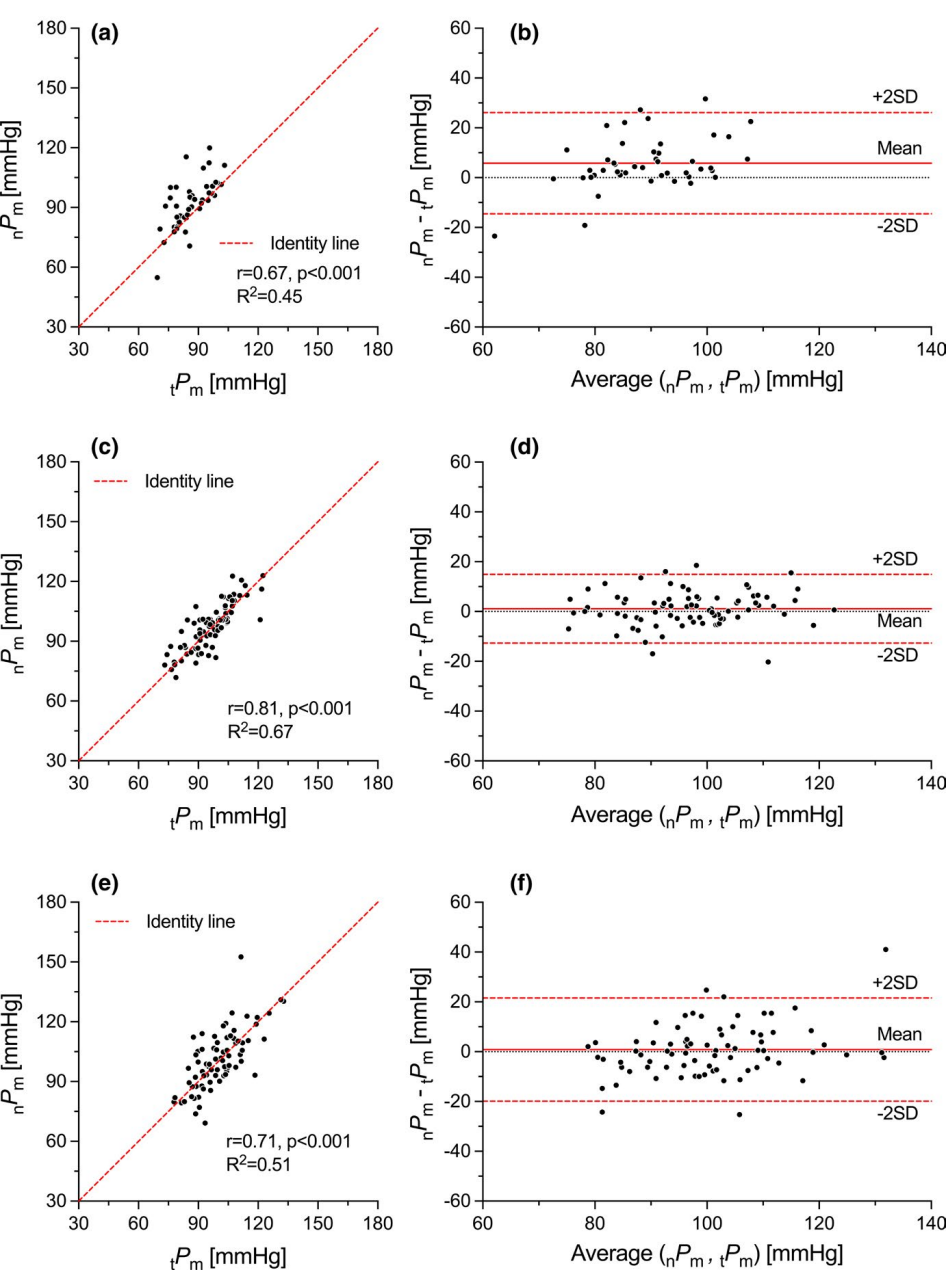
Figure S2—Correlation (a, c, and e) and Bland–Altman (b, d, f) plots between tonometer mean pressure (_t_ *P*_m_) and pressure estimated with the new technique (_n_ *P*_m_). (a, b) Young people (<35 years, *N* = 47); (c, d) middle‐aged people (35–59 years, *N* = 78); (e, f) older adults (≥60 years, *N* = 78)

As for *γ*, the pressure estimation using the new method performed better, on average, in middle‐aged and older adults; the average differences with tonometry for _n_ *P*_s_ and _n_ *P*_m_ were 1.8 (limits of agreement: −29.9 to 33.6) mmHg (*p* = 0.31) and 1.3 (limits of agreement: −12.6 to 15.2) mmHg (*p* = 0.11) in middle‐aged subjects, and −0.6 (limits of agreement: −42.8 to 41.6) mmHg (*p* = 0.80) and 0.3 (limits of agreement: −18.5 to 19.1) mmHg (*p* = 0.76) in older adults (Figure S2 (https://figshare.com/s/4aab7f7fd026d8fbb761) and Figure 4 for *P*_s_ and *P*_m_, respectively). However, as for γ, the interaction between age and _n_ *P*_s_ or _n_ *P*_m_ was not significant (*β* = −0.078, 95% CI [−0.260–0.104], *p* = 0.40 and *β* = −0.057, 95% CI [−0.198–0.084], *p* = 0.43, respectively). Further, the clinical background did not affect the pressure estimation (Table S2 https://figshare.com/s/4aab7f7fd026d8fbb761).

## 4 DISCUSSION

In this study, we proposed a new technique where the local wave speed, estimated by noninvasive local measurements of diameter distension and blood flow velocity waveforms, is used to estimate the parameters of an exponential function that allows converting the diameter waveform into a pressure waveform. In this proof of concept study, we compared the performance of the proposed method with CCA pressure measured using applanation tonometry in a group of healthy controls and hypertensive and diabetic patients. Results of the new technique compared well, on average, with those measured using applanation tonometry, but limits of agreements between the two techniques were high, especially for *P*_s_.

In the past two decades, several commercial TF‐based devices have been developed to estimate pressure noninvasively in the aorta. Although they are the most commonly used, their accuracy is still called into question. Ding et al. (2011) compared invasively measured central aortic pressure with estimates provided by two commercial devices, SphygmoCor and Omron HEM‐9000AI, both relying on the measurement of radial pressure waveforms calibrated with cuff measurement of brachial pressure. The first underestimated aortic *P*_s_ by 15 mmHg and the limits of agreement in the Bland–Altman plot were approximately −33 to 3 mmHg. The Omron device performed slightly better on average, with limits of agreement of approximately ±20 mmHg. Laugesen et al. (2014) showed that calibrating the radial pressure waveform with oscillometric brachial pressure did reduce, on average, the underestimation of the SphygmoCor synthesized central *P*_s_, but retained similarly wide limits of agreement (±22 mmHg). Only the calibration using invasively measured aortic *P*_s_ and *P*_d_ considerably improved the accuracy (±11 mmHg).

Compared to applanation tonometry measurements, our method overestimated *P*_s_ and *P*_m_ by 3.8 and 2.3 mmHg in the overall study population. Although multivariate regression analysis did not yield any significant interaction between noninvasive parameters and age, these differences were largely attributable to young subjects, where *P*_s_ and *P*_m_ were overestimated by ~11% and ~7%, respectively, while smaller and nonsignificant differences were found in middle‐aged and older adults. However, limits of agreement for *P*_s_ were wider than that reported for commercial devices (−36.8 to 38.0 mmHg in middle‐aged and older adults). These results might be due to the fact that the brachial pressure waveform was not acquired in our study. Conversely, _b_ *P*_m_ used for calibrating the tonometer pressure waveform was estimated using average *FF* previously reported for the brachial artery; *FF* = 0.43 (Segers et al., 2009). Here, carotid _t_ *FF*, determined from the acquired tonometer waveforms, averaged 0.45 but ranged between 0.36 and 0.57, and a similar variability was reported for the brachial artery (Grillo et al., 2020). Hence, neglecting the age‐dependence and inter‐subjects variability of the brachial *FF* likely affected the accuracy of the calibration of the tonometer pressure waveforms. Grillo et al. (2020) recently proposed an alternative method for the estimation of a subject‐specific brachial *FF* derived from _b_ *P*_d_ and gender. They showed that the method predicts age‐differences of brachial *FF* more effectively in middle‐aged and older adults. However, as conceded by the authors, the accuracy of the proposed formula remained sub‐optimal and unable to capture the high inter‐subject variability of the brachial *FF*. When applied to our data, estimation of _b_ *P*_m_ from _b_ *P*_d_ and gender did not improve the agreement between _n_ *P* and _t_ *P* in any of the age groups considered here.

The accurate estimation of *P*_s_ is undoubtedly important, however little attention is generally given to the shape and high frequency components of the pressure waveform synthesized from distal measurements. The pressure waveform at any arterial location is widely accepted to be the linear summation of the forward travelling pressure waves, generated by left ventricular contraction, and the backward travelling waves, originated at reflection sites when the forward travelling wave meets discontinuities (i.e., mismatched bifurcations and downstream tapering of the arterial tree) (Abdullateef et al., 2020; Khir & Parker, 2005). Given the complex structure of the arterial tree, the magnitude and timing of reflected waves are highly location‐dependent, making the estimation of pressure at any location from pressure acquired elsewhere in the arterial tree a complicated task. Indeed, the accuracy of estimating aortic waveform from radial measurements using transfer functions remains controversial. Segers et al. (2005) found that the augmentation index (AIx), an estimate of the relative magnitude of the reflected wave, calculated from the TF‐synthesized aortic pressure mildly correlated with the carotid AIx acquired using tonometry, although strongly correlated with that of the radial pressure waveform used in the transfer function. This result contradicts previous findings showing that carotid AIx strongly correlates with that of invasively measure aortic pressure waveforms (Chen et al., 1996), casting further doubts on the accuracy of generalized TF‐based aortic waveforms (Millasseau et al., 2003; Segers et al., 2005).

The magnitude and timing of reflected waves in central arteries has been positively associated with ventricular function (Park et al., 2020) and the incidence of cardiovascular events (Sugawara et al., 2009; Wang et al., 2010). Therefore, findings by Segers et al. (2005) suggest that the pressure waveform estimated via transfer function might carry information on reflections at peripheral sites but be less than ideal to evaluate the subject‐specific cardiac risk. Additionally, a previous study from our group showed good agreement between wave intensity analysis, using standard invasive *P* and *U* and noninvasive *D* and *U* methods (Li & Khir, 2011). This suggests that the information on the complex interaction between forward and backward waves is better captured by using local measurements of *U* together with either *P* or *D*, further supporting the new approach presented here; using the local ln*DU*‐loop to facilitate the estimation of local pressure waveform.

Methods that noninvasively estimate pressure from local arterial waveforms are likely to provide more accurate alternatives to transfer functions, following the rationale that local waveforms necessarily carry more representative information on local hemodynamics than peripheral pressure. Vennin et al. (2015) used the “water hammer” equation (Khir et al., 2001) to convert the upstroke of the flow velocity waveform into the pressure upstroke, and then modelled the elastic recoil in diastole with an exponential decay function and the pressure peak in late systole. The method yielded good results both in a one‐dimensional computational model of the arterial tree and in vivo. However, the use of invasively measured aortic *P* for the estimation of *c* using the sum of squares method (Davies et al., 2005) makes this technique less likely to be used clinically. Also, the accuracy of their technique remains to be examined when using a noninvasive estimation of *c*.

Beulen et al. (2011) were the first to use the relationship between *c* and arterial distensibility to convert the arterial diameter/area distension waveform into *P*. It is worth noting, however, that the underlying assumption in Beulen et al. is that *c* is constant in the investigated pressure range, yielding to a linear *P*–*D* ^2^ relationship. While such assumption is reasonable for the flexible tubes used for the validation of their method, arteries exhibit a nonlinear, approximately exponential relationship (Fung, 1967) and *c* is pressure‐dependent (Spronck et al., 2015). Application of their method on our data underestimated *P*_s_ (Figure S3 https://figshare.com/s/4aab7f7fd026d8fbb761). On the contrary, we assumed that *c* determined by the ln*DU*‐loop in early systole describes the slope of the *P*–*D* ^2^ relationship in the proximity of *P*_d_ but allows the estimation of *γ* that, together with local *D*_d_ and *P*_d_ (here assumed equal to *P*_d_ in the brachial artery), defines the exponential relationship between *P* and *D* ^2^. Hence, our method relaxes the assumption that *c* is pressure independent. As a result, the _n_ *FF* closely matched that calculated on the tonometer pressure acquisition (Table 1) and _n_ *P*_s_, on average, did not underestimate but agreed well with _t_ *P*_s_.

### 4.1 Limitations

As all the noninvasive methods for the estimation of pressure, whether based on transfer functions or local arterial waveforms, the accuracy of our method strongly depends on the fidelity of measurement of peripheral *P*, with brachial cuff measurement typically under and overestimating *P*_s_ and *P*_d_, respectively (Picone et al., 2017). Our method requires only *P*_d_ and, hence, is less affected by any potential inaccuracies pertaining cuff measurements. However, the inaccuracy of the measurements of brachial pressure likely affected the comparison between the two techniques, since tonometer waveforms were calibrated using both *P*_s_ and *P*_d_.

The accuracy of determining the local pressure waveform proposed in this work strongly depends on the accuracy of the estimation of wave speed *c* which appears squared in the formulas (Equation 9). Here, we used the ln*DU*‐loop method to noninvasively estimate *c* from the relationship between *D* and *U* in early systole. It was suggested previously that the accuracy of the loop methods is affected by the proximity to the reflection site and magnitude of the reflected waves (Borlotti et al., 2014; Segers et al., 2014) and that the ln*DU*‐loop method might underestimate *c* at the level of the CCA (Willemet et al., 2016). This underestimation was not observed here; *c* estimated with the ln*DU*‐loop and *D* ^2^ *P*‐loop (Alastruey, 2011), which is not affected by reflections, did not differ significantly (Giudici et al., 2021). Nevertheless, the method proposed here can be applied on any noninvasive estimate of *c*, provided that the correct *P_c_* is known.

## 5 CONCLUSIONS

This proof of concept study introduces a method for estimating pressure using local hemodynamic parameters recorded noninvasively. The proposed technique was tested on the common carotid artery where reference pressures for comparison could also be acquired noninvasively using tonometry. The promising result presented suggests that pressure can be estimated noninvasively at any arterial location where blood velocity and diameter waveforms can be acquired via ultrasound, making the measurement of central aortic pressure a real possibility. Doing so will characterize ventricular afterload more precisely, including potentially cardiovascular risk. Further work is warranted to test the effectiveness of the new method against invasively measured pressure and in estimating central aortic pressure.

## DISCLOSURES

JKC is a former president of the Artery Society. MK is responsible for clinical studies at Esaote SpA (Genova, Italy).

## AUTHORS CONTRIBUTION

AG contributed to the conceptualization, data analysis, manuscript drafting, and editing. AG and AWK developed the analytical method. AWK, CP, JKC, MK, and IBW contributed to the conceptualization, manuscript editing, and project supervision. CM contributed to the data acquisition and management.

## Supporting information



Supplementary Material
