# Supplementary material for: Transfer‐function‐free technique for the noninvasive determination of the human arterial pressure waveform
Source: Physiol Rep. 2021 Sep 22;9(18):e15040. doi: 10.14814/phy2.15040 (PMC8459031; doi:10.14814/phy2.15040)
Supplement: Supplementary file 1 — Supplementary Material [file PHY2-9-e15040-s001.docx]

**DATA SUPPLEMENT**

Table S1 – Clinical background and drug treatment of the population included in the study. Data are presented as “yes (%)”.

|  | **All**  **N=203** | $\boldsymbol{\leq}$**35 years**  **N=47** | **36-59 years**  **N=78** | $\boldsymbol{\geq}$**60 years**  **N=78** |
| --- | --- | --- | --- | --- |
| *Hypertension* | $92 (45\%)$ | $0 (0\%)$ | $35 (45\%)$ | $57 (73\%)$ |
| *Dyslipidemia* | $63 (31\%)$ | $0 (0\%)$ | $15 (19\%)$ | $48 (62\%)$ |
| *Type 1 diabetes* | $32 (16\%)$ | $17 (36\%)$ | $11 (14\%)$ | $4 (5\%)$ |
| *Type 2 diabetes* | $79 (39\%)$ | $0 (0\%)$ | $24 (31\%)$ | $55 (71\%)$ |
| *Antihypertensive* | $76 (37\%)$ | $0 (0\%)$ | $21 (27\%)$ | $55 (70\%)$ |
| *Oral antidiabetic* | $63 (31\%)$ | $0 (0\%)$ | $14 (18\%)$ | $49 (63\%)$ |
| *Insulin* | $61 (30\%)$ | $17 (36\%)$ | $19 (24\%)$ | $25 (32\%)$ |
| *Statin* | $69 (34\%)$ | $0 (0\%)$ | $22 (28\%)$ | $47 (60\%)$ |
| *Antiplatelet* | $24 (12\%)$ | $0 (0\%)$ | $5 (6\%)$ | $19 (24\%)$ |

Table S2 – Results of the multivariate regression analsys.

|  | ${}_{n}\gamma$ | | ${}_{n}{P_{m}}$ | | ${}_{n}{P_{s}}$ | |
| --- | --- | --- | --- | --- | --- | --- |
|  | $\beta$ | *p* | $\beta$ | *p* | $\beta$ | *p* |
| ${}_{t}\gamma$ | $0.767$ | $<0.0001$ | $-$ | $-$ | $-$ | $-$ |
| ${}_{t}{P_{s}}$ | $-$ | $-$ | $-$ | $-$ | $0.569$ | $<0.0001$ |
| ${}_{t}{P_{m}}$ | $-$ | $-$ | $0.814$ | $<0.0001$ | $-$ | $-$ |
| Age | $0.056$ | $0.47$ | $-0.057$ | $0.43$ | $-0.078$ | $0.40$ |
| T1DM | $-0.058$ | $0.29$ | $-0.032$ | $0.55$ | $-0.029$ | $0.69$ |
| T2DM | $-0.192$ | $0.007$ | $-0.09$3 | $0.16$ | $-0.107$ | $0.23$ |
| Antihypertensive | $-0.008$ | $0.91$ | $-0.032$ | $0.63$ | $-0.032$ | $0.72$ |
| Dyslipidemia | $0.025$ | $0.70$ | $0.018$ | $0.77$ | $0.023$ | $0.78$ |

${}_{n}\gamma$ : non-invasive estimated exponential parameter, ${}_{n}{P_{m}}$ : non-invasively estimated carotid mean blood pressure, ${}_{n}{P_{s}}$ : non-invasively estimated carotid systolic blood pressure, ${}_{t}\gamma$ : exponential parameter determined from the tonometer waveform, ${}_{t}{P_{m}}$ : tonometer mean blood pressure, ${}_{t}{P_{s}}$ : tonometer systolic blood pressure, T1DM : type 1 diabetes mellitus, T2DM: type 2 diabetes mellitus. T1DM, T2DM, antihypertensive and dyslipidemia were used as dichotomous variables (yes/no).

***
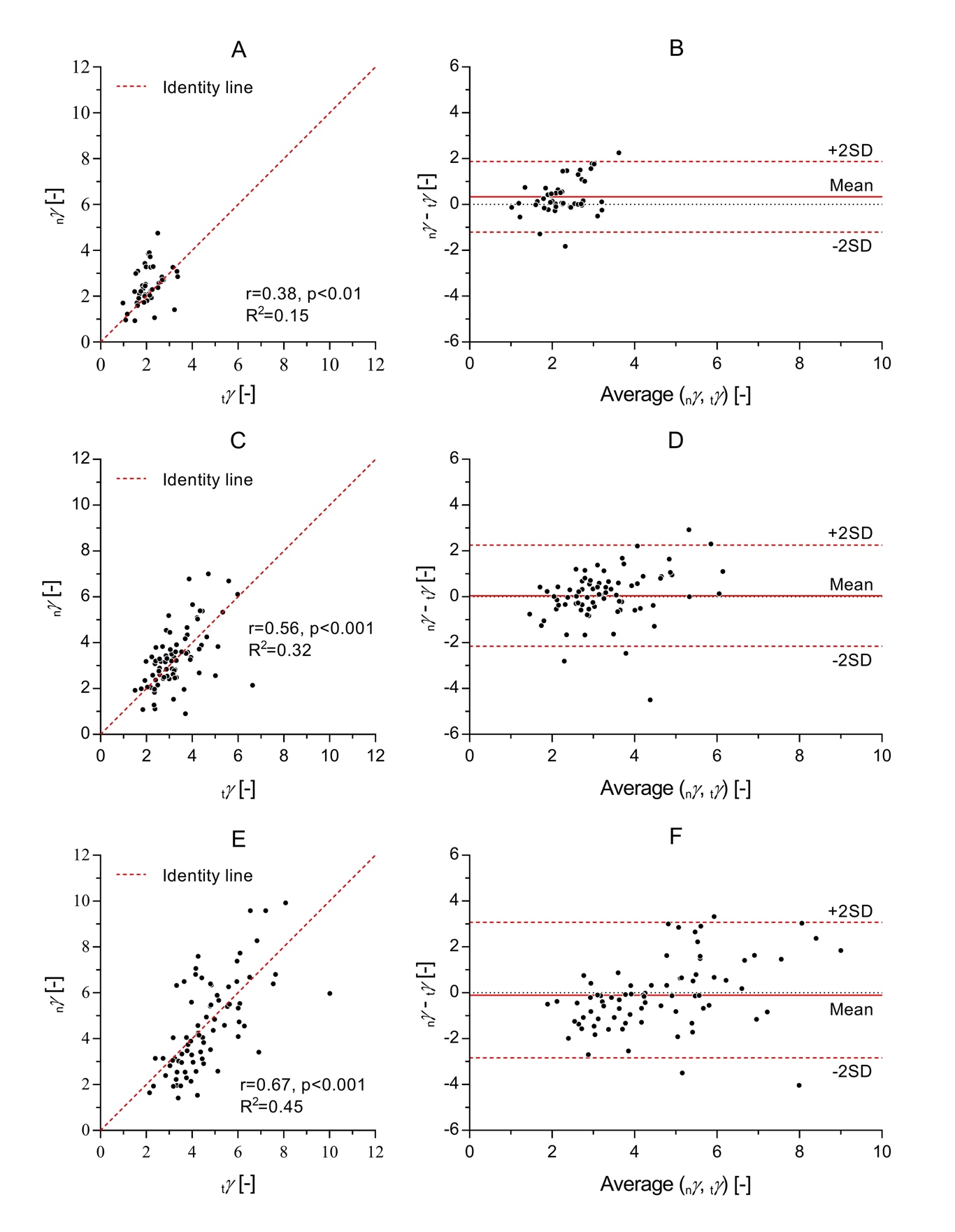
***

Figure S1 – Correlation (Panels A, C and E) and Bland-Altman (Panels B, D and F) plot between exponential constants estimated from the tonometer pressure, $\gamma$ (Eq. 10) , and from the non-invasive wave speed, ${}_{n}\gamma$ (Eq.9). Panels A and B: young people (<35 years); Panels C and D: middle-aged people (35-59 years); Panel E and F: older adults ($\geq$60 years).


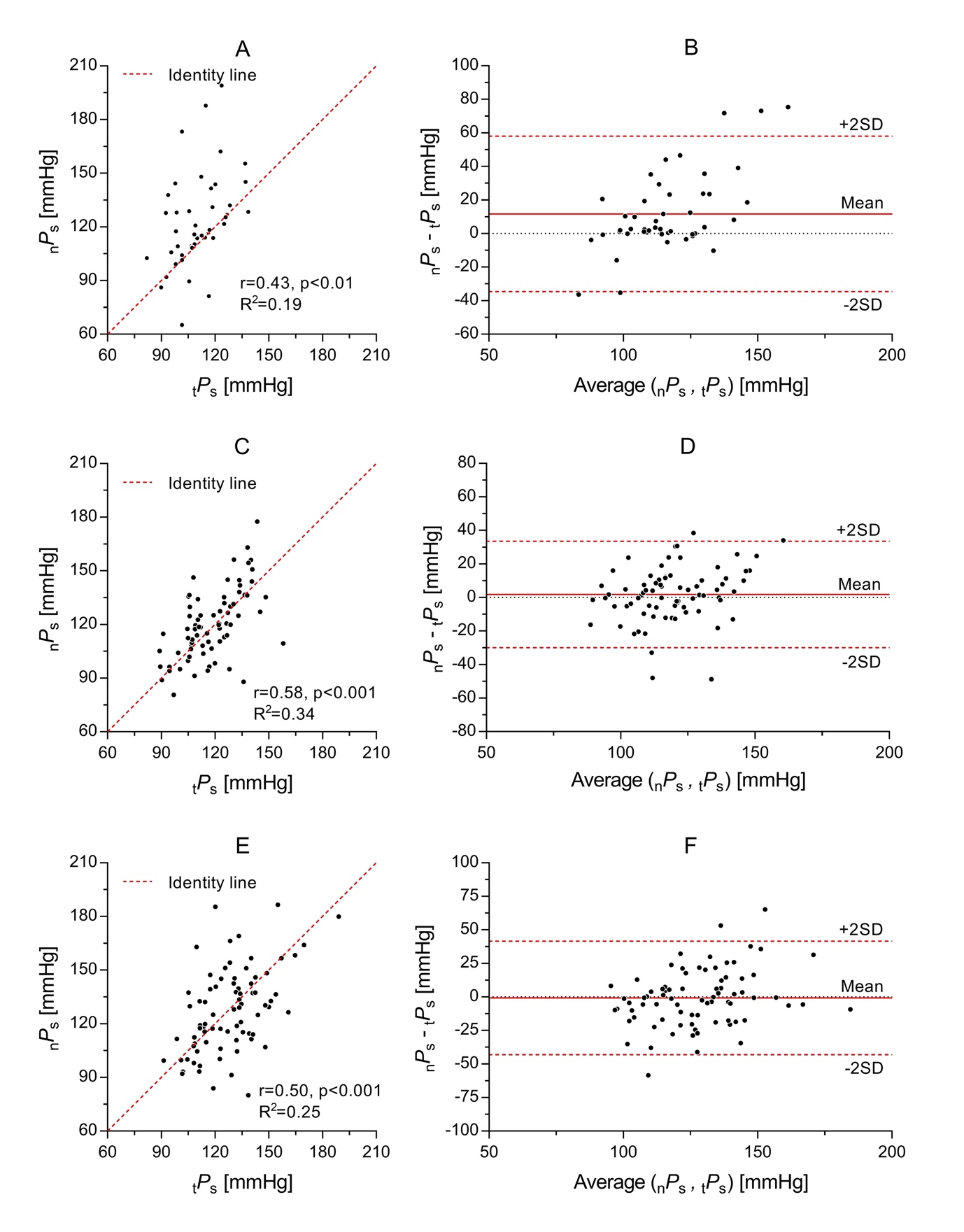


Figure S2 – Correlation (A, C and E) and Bland-Altman (B, D and F) plots between tonometer mean pressure (${}_{t}{P_{s}}$) and pressure estimated with the new technique (${}_{n}{P_{s}}$). Panels A and B: young people (<35 years); Panels C and D: middle-aged people (35-59 years); Panel E and F: older adults ($\geq$60 years).

**

**

Figure S3 – Comparison between the non-invasive method for the estimation of pressure proposed in this study (black line) and that of Beulen *et al.* (1) (red line). Panel A shows an example of pressure estimated at the common carotid artery applying the two methods to the ultrasound data of a subject of this study and Panel B the different nature of the underlying pressure-area relationship.

**References**

1. **Beulen BWAMM**, **Bijnens N**, **Koutsouridis GG**, **Brands PJ**, **Rutten MCM**, **Van de Vosse FN**. Towards noninvasive blood pressure assessment in arteries by using ultrasound. *Ultrasound Med Biol* 37: 788–797, 2011.
